# Supplementary material for: The genome sequence of an ichneumonid wasp, Gelis areator (Panzer, 1804)
Source: Wellcome Open Res. 2024 Nov 14;9:679. [Version 1] doi: 10.12688/wellcomeopenres.23342.1 (PMC12326167; doi:10.12688/wellcomeopenres.23342.1)
Supplement: Peer review history

# Reviewer report: Reviewer response for version 1

**DOI:** 10.21956/wellcomeopenres.25727.r128298

**Published:** 2025-09-04

**Authors:** Natalia Araujo

**Affiliations:** 1 Universite Libre de Bruxelles, Brussels, Brussels, Belgium

This is a manuscript report for the genome assembly of *Gelis aerator*. The species identity was validated using DNA barcoding from the COI region in addition to morphological identification, which is important for this taxonomically challenging group. The authors used the Darwin Tree of Life pipeline for sequencing PacBio HiFi and Hi-C reads from which the nuclear and mitochondrial genomes were assembled. This pipeline for sequencing and assembling genomes is well-established based on previous studies. All generated data is available in sequencing databases. The nuclear genome produced is of high quality, totaling 228.10 Mb, with over 98% of the assembly contained in 12 scaffolds. Additionally, 95.4% of BUSCO orthologs were reported as completed within the assembly. There are gaps in the assembly, but the number of Ns is low (0.03%). There is no information about the quality of the mitochondrial genome assembled, only its size is reported. The nuclear genome is not annotated. This is an adequate genome assembly report.

Are sufficient details of methods and materials provided to allow replication by others?

Yes

Is the rationale for creating the dataset(s) clearly described?

Yes

Are the datasets clearly presented in a useable and accessible format?

Yes

Are the protocols appropriate and is the work technically sound?

Yes

Reviewer Expertise:

Genomics, Bioinformatics and Evolution

I confirm that I have read this submission and believe that I have an appropriate level of expertise to confirm that it is of an acceptable scientific standard.

# Reviewer report: Reviewer response for version 1

**DOI:** 10.21956/wellcomeopenres.25727.r128297

**Published:** 2025-08-11

**Authors:** Danilo Trabudo do Amaral

**Affiliations:** 1 Universidade Federal do ABC Centro de Ciencias Naturais e Humanas (Ringgold ID: 425753), São Carlos, State of São Paulo, Brazil

1. The work presents a high-quality genome assembly with robust metrics and clear methodological documentation. I particularly appreciate the transparency in reporting the uncertainty in chromosome

 2. One possible improvement would be to complement this note with a brief diagnostic analysis, for example describing Hi-C contact patterns, repetitive content, or local heterozygosity in the affected region, and outlining an explicit action plan. 

 Potential follow-up steps could include:

 (i) local re-scaffolding of the region using YaHS without the --break parameter and testing alternative tools such as 3D-DNA or SALSA;

 (ii) performing a mini reassembly of the HiFi reads mapped to that window, followed by patching into chromosome 2; and

 (iii) comparing the result with Haplotype 2 to help distinguish between an assembly error and a true structural variation. This would further strengthen the assembly’s reliability and provide useful guidance for future work.

Are sufficient details of methods and materials provided to allow replication by others?

Yes

Is the rationale for creating the dataset(s) clearly described?

Yes

Are the datasets clearly presented in a useable and accessible format?

Yes

Are the protocols appropriate and is the work technically sound?

Yes

Reviewer Expertise:

Bioinformatics

I confirm that I have read this submission and believe that I have an appropriate level of expertise to confirm that it is of an acceptable scientific standard.

# Reviewer report: Reviewer response for version 1

**DOI:** 10.21956/wellcomeopenres.25727.r128305

**Published:** 2025-08-05

**Authors:** Sarah Inwood

**Affiliations:** 1 University of Otago, Dunedin, New Zealand

This article presents a high-quality genome assembly for the parasitoid wasp *Gelis areator. *This assembly was **generated from a single individual of unknown sex, using state-of-the-art methods, both in terms of sequencing (HiFi and Hi-C data) and assembly. This resulted in a chromosome-scale nuclear genome assembly as well as a mitochondrial genome assembly.

**Minor comments:**

 Discussion of the mitochondrial genome was very brief given it is ~6-9 kb longer than the reference sequences used. Was this expected? Is the larger size thought to be genuine or the result of misassembly? Was the assembly circularised?

 Brief discussion of cobionts/contaminants detected would have been interesting. Wolbachia was detected - is it known whether this species ever reproduces asexually? Was there any trace of viral genes in the genome, given many parasitoid wasps have endogenised viral genes/exogenous viral infections involved in parasitism?

Are sufficient details of methods and materials provided to allow replication by others?

Yes

Is the rationale for creating the dataset(s) clearly described?

Yes

Are the datasets clearly presented in a useable and accessible format?

Yes

Are the protocols appropriate and is the work technically sound?

Yes

Reviewer Expertise:

Genomics, host-parasite interactions, parasitoid wasps, insects, viruses, biocontrol

I confirm that I have read this submission and believe that I have an appropriate level of expertise to confirm that it is of an acceptable scientific standard.

# Reviewer report: Reviewer response for version 1

**DOI:** 10.21956/wellcomeopenres.25727.r113794

**Published:** 2024-12-21

**Authors:** Andrew Legan

**Affiliations:** 1 The University of Arizona Department of Entomology, Tucson, Arizona, USA

The article reports a 12 chromosome, 228 Mb ichneumonid wasp genome, generated from 86x PacBio Revio + Hi-C sequencing of a single female *Gelis areator*. Scaffold N50 of 19.4 Mb. The genome sequencing and assembly methods are state of the art and I offer no criticisms. I believe the genome assembly and associated article are scientifically sound contributions from the Darwin Tree of Life project.

Are sufficient details of methods and materials provided to allow replication by others?

Yes

Is the rationale for creating the dataset(s) clearly described?

Yes

Are the datasets clearly presented in a useable and accessible format?

Yes

Are the protocols appropriate and is the work technically sound?

Yes

Reviewer Expertise:

molecular ecology, comparative genomics, chemical ecology, behavior

I confirm that I have read this submission and believe that I have an appropriate level of expertise to confirm that it is of an acceptable scientific standard.
